# Supplementary material for: Single-photon detection and cryogenic reconfigurability in lithium niobate nanophotonic circuits
Source: Nat Commun. 2021 Nov 25;12:6847. doi: 10.1038/s41467-021-27205-8 (PMC8617300; doi:10.1038/s41467-021-27205-8)
Supplement: Supplementary file 1 — Supplementary Information [file 41467_2021_27205_MOESM1_ESM.pdf]

Supplementary Information

Single-photon detection and cryogenic reconfigurability in Lithium Niobate nanophotonic circuits

Emma Lomonte[1,2,3], Martin A. Wolff[1,2,3], Fabian Beutel[1,2,3], Simone Ferrari[1,2,3], Carsten Schuck[1,2,3], Wolfram H. P. Pernice[1,2,3,*], and Francesco Lenzini[1,2,3,*]

[1] *Institute of Physics, University of Muenster, 48149 Muenster, Germany*
[2] *CeNTech - Center for Nanotechnology, 48149 Muenster, Germany*
[3] *SoN - Center for Soft Nanoscience, 48149 Muenster, Germany*
** E-mail: wolfram.pernice@uni-muenster.de, lenzini@uni-muenster.de*

Supplementary Note 1 - Measurement of the nanowire absorption and reduced efficiency of the SNSPDs

To evaluate the absorption rate of our superconducting nanowires, we used a set of devices analogous to the one depicted in Supplementary Figure 1a. The device consists of two identical waveguides coupled via a directional coupler designed to exhibit a 50:50 splitting ratio. On one of the two waveguides we fabricated a nanowire with a length that was varied across several devices with a step of 8 μm. Grating couplers allow us to couple light in (inner couplers) and out (outer couplers) of the circuit. The absorption of the nanowires was measured by injecting laser light into one input of the directional coupler and comparing the power measured at the two outputs. As for the two SNSPDs fabricated at the outputs of the reconfigurable Mach-Zehnder interferometer, also these nanowires were covered with a ≃500 nm thick electrically-cured HSQ protection layer, which is approximately 35 μm wider than the nanowire from all sides (see Supplementary Figure 1a). This is used to prevent an irreversible damage of the nanowires during the RCA-1 cleaning process, employed in our fabrication workflow to remove the sputtered material that redeposits on the waveguide sidewalls during Ar etching of the LN film (see the Fabrication section in Methods for further details).

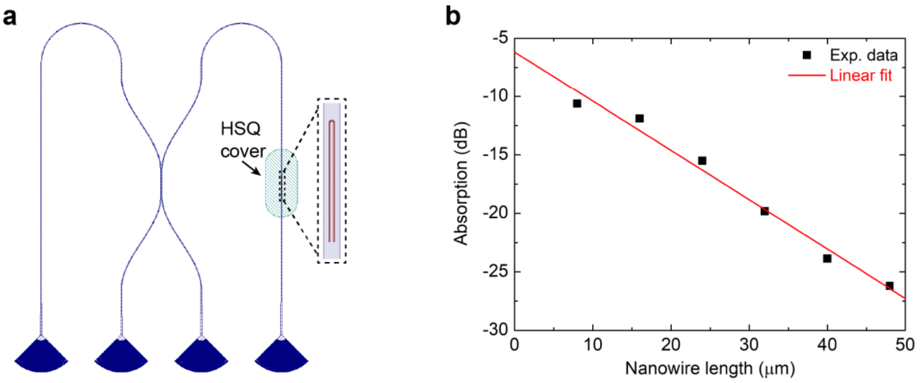

Supplementary Figure 1. **Measurement of the nanowire absorption. a** Schematic drawing of the device used for evaluating the absorption rate of the nanowire. The device consists of two identical waveguides, which are coupled via a 50:50 directional coupler. On one of the two waveguide, a U-shaped nanowire of varying length (here depicted in red) is fabricated and clad with a HSQ cover (here in green). **b** Measured absorption as a function of the nanowire length.

In Supplementary Figure 1b we report the measured absorption as a function of the nanowire length, plotted in a dB scale. The experimental data are fitted with a linear function defined as:

$$Absorption\ [dB] = -\alpha x + B, \qquad (1)$$

where x is the length of the nanowire, B is the intercept between the curve and the vertical axis, and α the slope of the function. From the fit we found $\alpha = (0.42 \pm 0.04)$ dB/µm, and $B = -(6.2 \pm 1.2)$ dB. We note that a negative coefficient B implies a large insertion loss (≃6 dB) of the waveguide section covered by the HSQ layer even for a null nanowire length. Such insertion loss is not calibrated out in our estimation of the SNSPDs on-chip detection efficiency. Due to the overall dimensions of the HSQ cover, an additional propagation loss of ≃0.1 dB/µm in proximity of the detector region can be estimated. Upon subtracting this contribution from the measured coefficient α we can infer an absorption rate of the superconducting nanowires ≃ 0.32 dB/µm, a value in good agreement with the prediction of our numerical simulations (0.35 dB/µm).
We attribute the reduced efficiency of our SNSPDs to this lossy behaviour of the waveguide in the detector region covered by the HSQ layer, and identify two possible causes: *i)* the presence of small residuals of the NbTiN film after dry etching, which are not large enough to short the nanowires and affect their operation, but can induce substantial absorption loss on the propagating light; *ii)* the redeposition of sputtered material on the waveguide sidewalls, which causes a large scattering effect on the propagating light. Such large propagation loss cannot be present in the remaining optical circuit, where both sidewall redeposition and potential residuals of the NbTiN film are removed during the RCA-1 cleaning bath. We expect that in future experimental implementations the efficiency of our detectors can be improved via an optimization of the etching time of the superconducting film, or by adiabatically increasing the width of the waveguide in order to reduce the overlap between the optical mode and the rough waveguide sidewalls.

Supplementary Note 2 - Electrical crosstalk

To model electrical crosstalk between the EOM and SNSPDs channels, we make use of the electric circuit schematically depicted in Supplementary Figure 2a. A low noise amplifier (LNA) with an input load $R_L = 50\ \Omega$ is used to amplify the bias current diverted to the readout of the circuit (either a time tagger or a digital oscilloscope) upon the absorption of a single photon by the superconducting nanowire[45]. The SNSPD, when in its superconducting state, is approximated as a circuit element with zero resistance and a kinetic inductance L_K. The value of L_K can be estimated from the decay time of the SNSPD signal (see Fig. 3c,d) via the relation $\tau = L_K/R_L$[45], and is found to be equal to ≃300 nH for our detectors.
Crosstalk due to capacitive coupling is approximated in the circuit as a voltage source with a magnitude equal to the voltage applied to the EOM (V_{EOM}), which is coupled to the RF line connecting the SNSPD to the bias tee via a fictitious capacitor C. Effectively, this results in a current source $I_{NOISE} = C\, dV_{EOM}/dt$ in parallel with the 50 Ω load of the LNA and the SNSPD, which is characterized by a complex impedance Z_{DET} growing linearly with the modulation frequency f of the EOM. In Supplementary Figure 2b we report the values of the total noise current I_{NOISE} generated by capacitive coupling (black curve), of the current directed to the SNSPD (red curve), and of the current directed to the readout (blue curve), calculated with the electric circuit model of Supplementary Figure 2a. Calculations are performed in the frequency range $f = 1$ MHz ÷ 1 GHz for $L_K = 300$ nH, and the plotted data normalized to the maximum value of I_{NOISE} ($= C 2\pi f_{max}|V_{EOM}|$). At low modulation frequencies, when $|Z_{DET}| \ll R_L$, most of the noise current is directed to the SNSPD, and $I_{SNSPD} \simeq I_{NOISE}$. Conversely, as the modulation frequency approaches ≃ 100 MHz and $|Z_{DET}|$ becomes much larger than R_L, most of the noise current is directed to the readout. Correspondingly, I_{SNSPD} saturates to a steady value, regardless of the increasing modulation frequency. Importantly, this result suggests that SNSPDs are naturally resilient to high frequency noise caused by electrical crosstalk, and, upon filtering out the noise current directed to the readout of the circuit, their normal functionality can be recovered.
To verify the validity of our model, in Supplementary Figure 2c we show the ratio $V_{Readout}/V_{EOM}$ measured for Det2 in the same frequency range. This measurement was performed by driving the EOM with a small-amplitude RF signal leaving from Port1 of a vector network analyzer (VNA), and by connecting Port2 to the output of the LNA. The reported data are the measured S_{21} parameter corrected by the gain factor of the amplifier and plotted in a dB scale. The black trace plotted in the figure was obtained by biasing the SNSPD with a current larger than its critical current, in order to break its superconductivity. When in a resistive state, our detectors are characterized by a large normal resistance ≃1.5 MΩ, and thus the noise current generated by capacitive coupling is fully diverted to the readout of the circuit. Instead, the blue curve is acquired for a null current applied to the SNSPD,

such that the detector is in the superconducting state. As correctly predicted by our model, the blue curve falls below the black trace at low modulation frequencies and almost perfectly overlaps with it for $f > 100$ MHz.

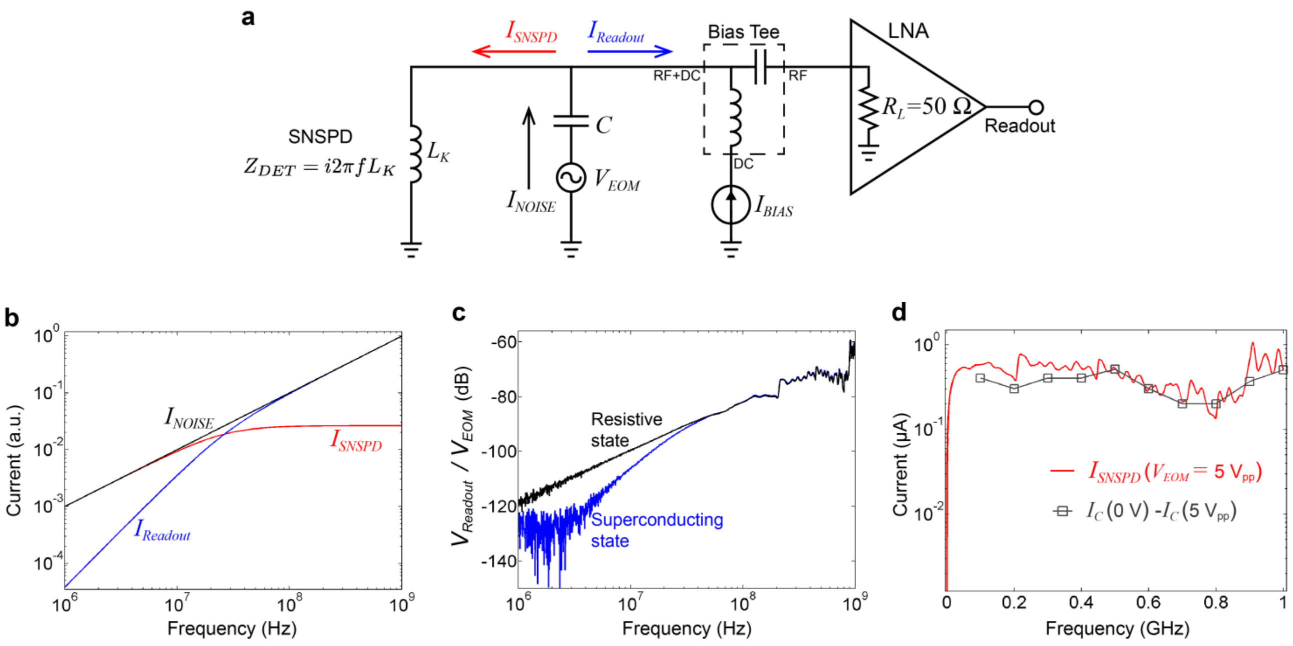

Supplementary Figure 2. **Electrical crosstalk between EOM and SNSPDs channels. a** Schematic drawing of the electric circuit used to model crosstalk due to capacitive coupling. The LNA is implemented in practice in our experiment using two amplifiers in series (ZFL-1000LN+, from Mini-Circuits) with a bandwidth of 0.1-1000 MHz. **b** Total noise current (black curve) generated by capacitive coupling, noise current directed to the readout of the circuit (blue curve), and noise current directed to the SNSPD (red curve), calculated with the electric circuit model of Supplementary Figure 2a. The plotted data is normalized to the maximum value of I_{NOISE}. **c** Ratio between the rms voltage measured at the readout of the circuit of Det2 (corrected by the gain factor of the amplifier) and the rms voltage applied to the EOM. The black trace is obtained by applying to the SNSPD a bias current larger than its critical current, in order to break its superconducting state. Voltage ratios < -130 dB fall below the sensitivity level of the employed VNA, resulting in a constant plateau at low frequencies for the blue trace. **d** Red curve: noise current delivered to the SNSPD for a voltage of 5 V_{pp} applied to the EOM, calculated from the data of Supplementary Figure 2c. Grey squares: difference between the critical currents of Det2 measured for a zero applied voltage to the EOM and an applied voltage of 5 V_{pp}. Measurements are performed at steps of 100 MHz in the frequency range $f = 0.1 \div 1$ GHz.

From the data plotted in Supplementary Figure 2c and the circuit model of Supplementary Figure 2a, we can calculate the noise current directed to the SNSPD for all frequencies through the relation:

$$I_{SNSPD} = I_{Readout} \frac{R_L}{|Z_{DET}|}. \qquad (2)$$

For the case of a driving voltage equal to the V_π of the EOM (≃18 V), we estimate that above 100 MHz I_{SNSPD} saturates to a root mean square (rms) value close to 1.5 μA, leading to a large variation of the on-chip detection efficiency during electro-optic modulation, and preventing to operate the detector close to its critical current. In contrast, for a driving voltage of 5 V_{pp} a lower current noise of ≃0.4 μA is inferred, which is a value that allowed us to explicitly show electro-optic modulation at frequencies up to 1 GHz (see Fig. 5b,c).

A direct measurement of the noise current delivered to Det2 was performed by comparing the critical current of the detector in case of zero voltage applied to the EOM, namely I_C(0 V), with the one measured for an applied voltage of 5 V_{pp}, I_C(5 V). Indeed, for modulation frequencies above 100 MHz, any noise exceeding the critical current of the detector caused latching of the nanowire into a permanent resistive state, and the effect of electrical crosstalk was directly observed as a reduction of the measured critical current. The measured data (grey squares) are plotted in Supplementary Figure 2d as a function of the modulation frequency applied to the EOM, and display excellent agreement with the prediction of our circuit model (red curve).

We note that for modulation frequencies close to 1 GHz and a driving voltage > 10 V_{pp} effects of thermal crosstalk also started to become observable, and the critical current of the detector was found to decrease quadratically as a function of the applied voltage. The challenge of thermal crosstalk was not extensively investigated, but we argue that it could be addressed in several ways, e.g., by enlarging the spatial separation between EOM and SNSPDs, or by reducing the resistance of the electro-optic phase-shifter using wider and thicker electrodes. Alternatively, for high-speed modulation in the ~GHz regime, the length of the modulator can be increased in order to achieve a lower half-wave voltage.

A first reduction of electrical crosstalk can be readily achieved in future experiments by increasing the spatial separation between the SNSPD and EOM channels. As an example, Supplementary Figure 3b shows a S_{21} measurement performed with a VNA between the channels 1-7 of our RF probe (see Supplementary Figure 3a). In practice, this would correspond to a situation where SNSPD and EOM contact pads are placed at distance of ≃1.2 mm from each other. Although the reported measurements are performed without making contact on any metal pad, crosstalk due to capacitive coupling on-chip can be considered negligible at such a large spatial separation between the RF channels. A direct comparison of the plotted data with the black trace of Supplementary Figure 2c shows an enhanced isolation between RF channels of around 20 dB at all frequencies. We estimate that such a configuration would enable high-speed modulation at the full half-wave voltage of the EOM, with a noise current I_{SNSPD} generated by electrical crosstalk below 0.2 μA.

Further improvement might be achieved by separating the EOM and SNSPDs driving channels onto different RF probes. In Supplementary Figure 3c we present a photograph of a double-probe setup mounted on a custom-made holder compatible with our cryostat. A S_{21} measurement performed with a VNA between the two probes (see Supplementary Figure 3d) indicates an isolation between RF channels below ≃-110 dB at all frequencies. Thus, such configuration would enable high-speed modulation with a negligible influence of electrical crosstalk on the detectors, and also eliminate the need of employing frequency filters at the output of the low noise amplifier.

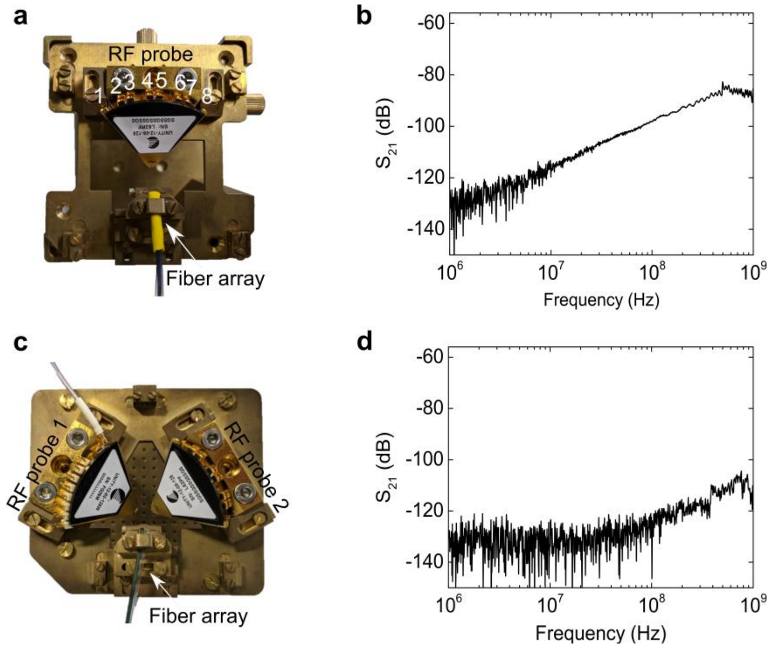

Supplementary Figure 3. **Improvement of electrical crosstalk in future experiments. a** Photograph of the standard setup employed to show the combined operation of EOM and SNSPDs. Fiber array and RF probe are mounted on a custom-made brass holder, which is placed inside the inner chamber of our closed-cycle cryostat. The RF probe (Unity Probe, from FormFactor) consists of 8 RF channels with a 125 μm separation between adjacent pins. **b** S_{21} measurement performed with a VNA between the channels 1-7 of the RF probe. Measurements are performed by driving channel 1 with a small-amplitude RF signal leaving from Port1 of the VNA. The signal at the output of channel 7 is amplified with the same LNA employed for the SNSPDs and connected to the Port2 of the VNA. The plotted data are corrected by the gain factor of the amplifier. **c** Double-probe setup for enhancing the isolation between EOM and SNSPDs channels. Fiber array and two identical RF probes are mounted on a custom-made brass holder compatible with our closed-cycle cryostat. **d** S_{21} measurement performed with a VNA between channel 1 of RF probe 1, and channel 4 of RF probe 2. Measurements are conducted with the same procedure explained in the caption of Supplementary Figure 2c.
